# Marine fungal metabolite butyrolactone I prevents cognitive deficits by relieving inflammation and intestinal microbiota imbalance on aluminum trichloride-injured zebrafish

**DOI:** 10.1186/s12974-022-02403-3

**Published:** 2022-02-07

**Authors:** Yingying Nie, Jingming Yang, Longjian Zhou, Zhiyou Yang, Jinyue Liang, Yayue Liu, Xiaoxiang Ma, Zhongji Qian, Pengzhi Hong, Allan V. Kalueff, Cai Song, Yi Zhang

**Affiliations:** 1 College of Food Science and Technology, Guangdong Provincial Key Laboratory of Aquatic Product Processing and Safety, Guangdong Provincial Engineering Laboratory for Marine Biological Products, Research Institute for Marine Drugs and Nutrition, Shenzhen Institute of Guangdong Ocean University, Guangdong Ocean University, Zhanjiang, 524088 China; 2 Collaborative Innovation Center of Seafood Deep Processing, Dalian Polytechnic University, Dalian, 116034 China; 3 School of Pharmaceutical Science and Technology, Tianjin University, Tianjin, 30072 China; 4 College of Pharmaceutical Sciences and Chinese Medicine, Southwest University, Chongqing, 400715 China; 5 Ural Federal University, Ekaterinburg, 620002 Russia; 6 Institute of Translational Biomedicine, St. Petersburg State University, Saint Petersburg, 199034 Russia

**Keywords:** Inflammation, Oxidative stress, Butyrolactone I, Neurodegenerative diseases, Acetylcholinesterase, Intestinal flora

## Abstract

**Background:**

Mounting evidences indicate that oxidative stress, neuroinflammation, and dysregulation of gut microbiota are related to neurodegenerative disorders (NDs). Butyrolactone I (BTL-I), a marine fungal metabolite, was previously reported as an in vitro neuroprotectant and inflammation inhibitor. However, little is known regarding its in vivo effects, whereas zebrafish (*Danio rerio*) could be used as a convenient in vivo model of toxicology and central nervous system (CNS) diseases.

**Methods:**

Here, we employed in vivo and in silico methods to investigate the anti-NDs potential of BTL-I. Specifically, we established a cognitive deficit model in zebrafish by intraperitoneal (i.p.) injection of aluminum trichloride (AlCl_3_) (21 μg) and assessed their behaviors in the T-maze test. The proinflammatory cytokines interleukin-1β (IL-1β) and tumor necrosis factor-α (TNF-α) as well as acetylcholinesterase (AChE) activity or glutathione (GSH) levels were assayed 24 h after AlCl_3_ injection. The intestinal flora variation of the zebrafish was investigated by 16S rDNA high-throughput analysis. The marine fungal metabolite, butyrolactone I (BTL-I), was used to modulate zebrafish cognitive deficits evoked by AlCl_3_ and evaluated about its effects on the above inflammatory, cholinergic, oxidative stress, and gut floral indicators. Furthermore, the absorption, distribution, metabolism, excretion, and toxicity (ADMET) and drug-likeness properties of BTL-I were studied by the in silico tool ADMETlab.

**Results:**

BTL-I dose-dependently ameliorated AlCl_3_-induced cognitive deficits in zebrafish. While AlCl_3_ treatment elevated the levels of central and peripheral proinflammatory cytokines, increased AChE activity, and lowered GSH in the brains of zebrafish, these effects, except GSH reduction, were reversed by 25–100 mg/kg BTL-I administration. Besides, 16S rDNA high-throughput sequencing of the intestinal flora of zebrafish showed that AlCl_3_ decreased Gram-positive bacteria and increased proinflammatory Gram-negative bacteria, while BTL-I contributed to maintaining the predominance of beneficial Gram-positive bacteria. Moreover, the in silico analysis indicated that BTL-I exhibits acceptable drug-likeness and ADMET profiles.

**Conclusions:**

The present findings suggest that BTL-I is a potential therapeutic agent for preventing CNS deficits caused by inflammation, neurotoxicity, and gut flora imbalance.

**Supplementary Information:**

The online version contains supplementary material available at 10.1186/s12974-022-02403-3.

## Background

Neurodegenerative disorders (NDs), such as Alzheimer’s disease (AD) and Parkinson’s disease (PD), are chronic, progressive, and severely debilitating neurological disorders [1, 2]. NDs are characterized by cognitive and motor deficits, accompanied by neuronal apoptosis and reduced neurotransmission [3, 4]. Oxidative stress and inflammation play critical roles in neuronal apoptosis [5–9] and may, thus, be considered potential risk factors for NDs. Various factors, including peripheral or brain inflammation, β-amyloid peptide (Aβ), pathogenic infection and toxins (e.g., aluminum), activate brain microglial cells [10–12]. The release of proinflammatory cytokines and reactive oxygen species (ROS) trigger and amplify damage to neurons and astrocytes, whereas oxidative stress and inflammation further promote neuroinflammation, which in turn activates microglia, eventually impairing neurons and astrocytes [5, 7, 12–14]. Oxidative stress and inflammation are involved in cyclin-dependent kinase 5 (CDK 5) activation-induced Tau hyperphosphorylation, resulting in neurofibrillary tangles, another important pathological marker of AD [5].

In addition, an increasing number of studies have shown that intestinal microorganisms are closely related to the occurrence of NDs and their mechanism also involves inflammation, oxidative stress and neurotransmitters [15, 16]. Researchers revealed that the increase of proinflammatory gut microbiota (GMB) taxon (the producers of liposaccharides) and reduction of anti-inflammatory GMB taxon are possibly associated with the peripheral inflammation in patients with impaired cognition and brain amyloidosis. The GMB may influence the cognition by changing gut permeability and inflammatory-oxidative stress state, producing short-chain fatty acids and neurotransmitters, promoting or reducing beta-amyloid deposition and transfer to brain, etc. [17]. Thus, GMB is becoming a new target of ND treatment.

**Fig. 1:**
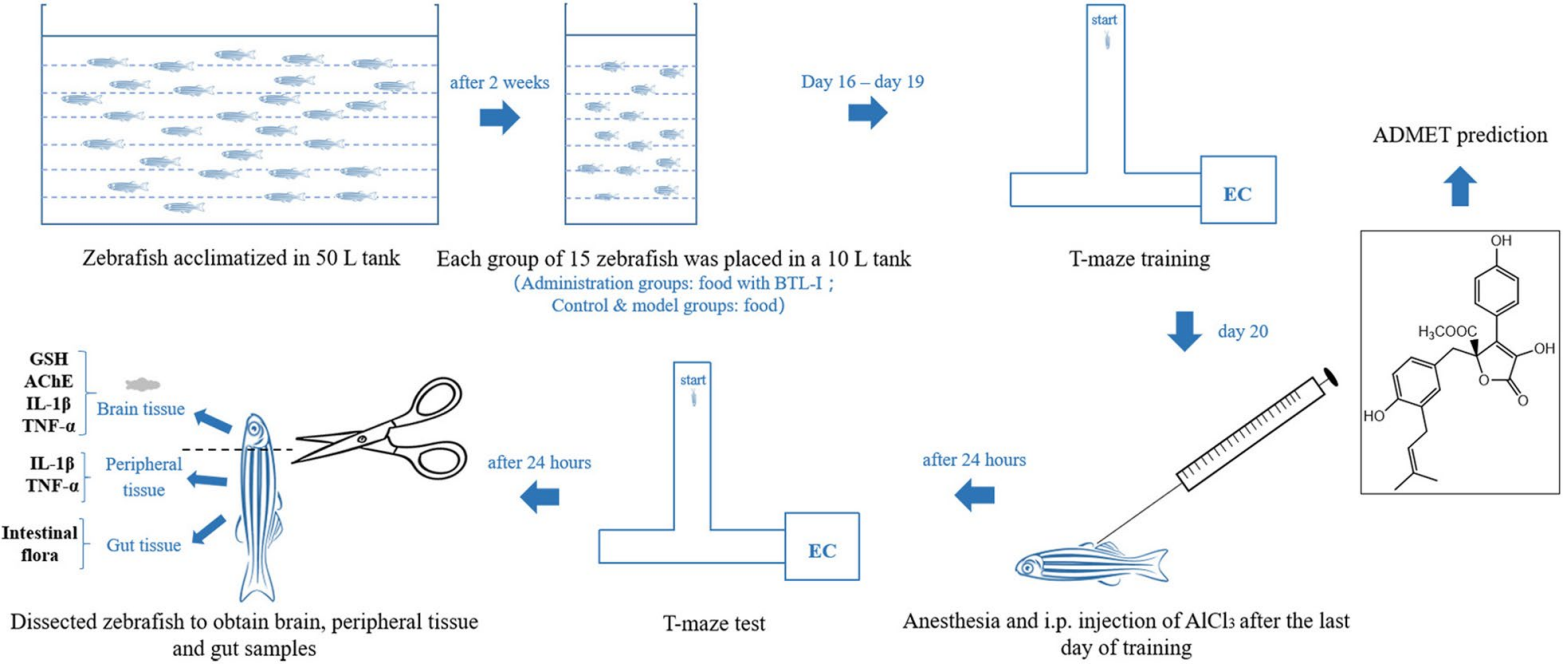
A general diagram summarizing the experimental design used in the present study. Inset: the chemical structure of BTL-I

Notably, current clinical drugs for the prevention and treatment of NDs manifest limited efficacy. For example, donepezil partially relieves symptoms of AD without reversing or preventing its progression [5]. The poor understanding of NDs pathogenesis restricted new drug development [18, 19]. The repeated failure to develop anti-AD drugs targeting orphan targets, such as Aβ and Tau, may be substantially related to malignant amplification induced by neuroinflammation and oxidative stress. Thus, inhibiting inflammation and oxidative stress and regulating imbalanced GMB to protect neurons and intervene in the early stage of disease has become an important new strategy in developing novel anti-NDs agents [20]. Aryl butyrolactones (BTLs), including butyrolactone I (BTL-I, Fig. 1 inset), are characteristic natural products of fungi (e.g., *Aspergillus* sp. and *Penicillium* sp.) [21, 22]. Our previous studies have shown that BTL-I has strong in vitro anti-neuroinflammatory effects, inhibiting LPS-induced inflammatory proliferation of microglia, the release of inflammatory mediators (NO and IL-1β) and ROS, the expression of the inflammatory target enzyme cyclooxygenase-2 (COX-2), and intracellular migration of the signaling protein NF-κB p65 [23]. Moreover, BTL-I plays a versatile anti-neurodegenerative role through multiple mechanisms, such as neuronal nutrition and inhibition of neuronal injury [24–27]. However, since previous BTL-I studies were limited to in vitro studies, its in vivo effects remain to be elucidated as well as its influence on GMB.

Due to their high genetic and physiological homology to humans, zebrafish has long been used as a powerful in vivo model to assess anti-inflammatory drugs [28, 29] and neuronal injury [30]. Zebrafish possess innate and acquired immune systems similar to those of mammals [31] display well-characterized learning, memory, addiction and other behaviors that correspond to clinical phenotypes, and host rich GMB in their intestine [32], enabling them an ideal model for our purpose [33–36].

AlCl_3_ causes AD-like pathology, aggravating neuroinflammation, oxidative stress and AChE activity in the rodent brain [12, 37, 38]. The effect of AlCl_3_ on zebrafish cognition remains unclear [39]. In the present study, we established a neurotoxic zebrafish model (induced by AlCl_3_) to assess the potential effects of BTL-I on memory and cognitive impairment in vivo. The putative neuroprotective activity of BTL-I in zebrafish was further investigated by evaluating AChE activity and GSH levels in the brain and by detecting the levels of the inflammatory cytokines IL-1β and TNF-α in both central and peripheral tissues. The 16S rDNA high-throughput method was used to determine the structure and changes of zebrafish intestinal flora. The in silico tool ADMETlab was further applied to evaluate the absorption, distribution, metabolism, excretion, and toxicity (ADMET) and drug-likeness properties of BTL-I.

## Methods

### Animals and model development

Adult wild-type AB zebrafish (approximately 6–8 months old; 50:50% male:female ratio) used in the present study were obtained from a commercial supplier (Shanghai Jiayu Aquarium, Shanghai, China) and acclimatized in a 50-L aquarium in the aquatic facility of Guangdong Ocean University for at least 2 weeks. The fish were kept on a 14 h:10 h light:dark cycle (lights on at 7 am) at a temperature of 25 ± 2 °C in a recirculating tank system. The zebrafish were maintained according to standard conditions [40] and fed *Artemis* larvae twice a day at 9 am and 2 pm.

As shown in Fig. 1, to establish an AlCl_3_ model, 75 zebrafish (3.0 ± 0.4 cm in length) were randomly divided into control and AlCl_3_- and BTL-I-treated groups (*n* = 15 per group). BTL-I (25, 50 or 100 mg/kg/day) was administered with food for 20 days. The control and AlCl_3_ groups were fed equal amounts of normal food. Twenty days later, the AlCl_3_ and BTL-I groups were anesthetized and injected (i.p.) with AlCl_3_ solution (4.2 mg/mL, 5 μL, pH = 5.0 ± 0.2) using a 10-μL gas phase injection needle with a 0.5-mm outer diameter. The control group was injected with the same amount of saline. Memory testing was performed 24 h later.

Briefly, following a 24-h fasting period, the fish were anesthetized and then received an i.p. injection. For this purpose, eugenol was dissolved in 100 mL of anhydrous ethanol to prepare a 1 mg/mL stock solution that was added to 5 L of water (28 ± 1 °C) and stirred evenly. Zebrafish were then group-exposed to the anesthetic, and after stopping swimming (immobile > 2–3 min), they were quickly injected with AlCl_3_. After the injection, the animals recovered in a water-containing beaker and returned to the holding tanks once their normal swimming resumed [41].

### T-maze behavioral testing

The aquatic T-maze was used for cognitive testing, as described previously, with modifications [42, 43]. The maze comprised a long vertical arm (50 cm) and two short horizontal arms (20 cm), with an arm width of 10 cm, a depth of 10 cm, and a water depth of 8 cm. The right arm was connected to a rectangular water tank (22 cm × 20 cm × 15 cm) with a black outer wall; sand and stones were added to the bottom of the tank, and bait was set inside the tank to provide an enriched chamber (EC) (Fig. 1). During the final 4 days of treatment, 6 fish were randomly selected from each group, and the fish were individually trained for 5 min daily to locate the EC zone. If a fish did not enter the EC zone within the 5-min training session, it was guided into the EC zone and kept there for > 30 s. Following 4 days of training, the trained fish were placed individually one day later into the starting area of the long arm for behavioral testing, with scoring of the latency time (s) to enter the EC zone and stay there for > 30 s. If a fish did not enter the EC zone within the 5-min test, the latency time was recorded as 300 s. Behavioral testing was performed between 10 am and 1 pm. A Microsoft LifeCam Studio 1080p HD camera was used to record videos with Apowersoft software (Apowersoft Co. Ltd., Hong Kong, China). Supersys software was used for off-line video analyses (Shanghai Xinruan Information Technology Co. Ltd., Shanghai, China), assessing the latency of the first entry into the EC zone(s), the average swimming speed (cm/s), and the number of EC entries.

### Reagents

BTL-I was isolated for this study from the marine fungus *Aspergillus terreus* C23-3 as described previously [23]. The BCA protein, GSH and AChE assay kits, fish IL-1β enzyme-linked immunosorbent assay (ELISA) kit and fish TNF-α ELISA kit were purchased from Nanjing Jiancheng Bioengineering Institute (Nanjing, China). Eugenol and AlCl_3_ were purchased from Huaxia Reagent (Chengdu, China) and Xiya Reagent (Shandong, China), respectively.

### Molecular biomarker assays

Twenty-four hours after behavioral testing, the fish were euthanized. Considering that the available assay kits could not measure the small tissue samples of individual fish, the 15 fish in each group were randomly divided into 3 subgroups, and samples of brain, peripheral and intestinal tract tissue from each subgroup (5 fish) were collected and combined immediately and freeze-dried at − 80 °C. All the samples (except for the gut samples) were homogenized in phosphate-buffered solution (PBS) for further assays. The supernatant was collected by centrifugation at 252 g at 4 °C for 15 min. Zebrafish brain sample supernatants were used to determine GSH levels and AChE activity. Moreover, zebrafish brain samples and peripheral tissue supernatants were also used to determine the levels of IL-1β and TNF-α [44] following the manufacturer’s instructions. The results are expressed as U of AChE/mg of protein and μmol of GSH/g of protein. Regression equations for the IL-1β and TNF-α standard curves were calculated according to the OD value, and logistic curves (4 parameters each) were used as the fitting models.

### Statistical analysis for behavioral and molecular biomarkers

Statistical analysis was performed by one-way analysis of variance (ANOVA) followed by a post hoc Dunnett test. The results are expressed as the mean ± SD. *P*-values < 0.05 indicated statistical significance for all tests.

### Gut flora sequencing and data analysis

Genomic DNA was extracted by protease K lysis. The variable region of the 16S ribosomal RNA gene V3–V4 was amplified by PCR, and the specific primer sequences were as follows: 357F 5′-ACTCCTACGGRAGGCAGCAG-3′ and 806R 5′-GGACTACHVGGGTWTCTAAT-3′. Bidirectional sequencing was performed according to Illumina high-throughput sequencing requirements, and the library was constructed by two-step PCR amplification. The PCR conditions were 94 °C for 2 min, 94 °C for 30 s, 56 °C for 30 s, 72 °C for 30 s (the primary PCR amplification 30 cycles, the secondary PCR amplification 8 cycles), 72 °C for 5 min, and a final extension at 10 °C. PCR amplification products were recovered by 2% agarose gel electrophoresis. Recycling was performed using an AxyPrepDNA gel recovery kit from Axygen.

The PCR-amplified products of the zebrafish gut samples were sequenced for 16S rDNA using the Illumina-Misq high-throughput sequencing platform [TinyGene Bio-Tech (ShangHai) Co., Ltd, China], and the sequence length was 450 bp. The raw data obtained from sequencing were evaluated for quality and optimized. Trimmomatic was used for sequence filtration, and FLASH was used for splicing. Ambiguous, homologous and some chimeras produced in the PCR process were subsequently screened using Mothur V.1.39.5 to obtain optimized sequences for subsequent operational taxonomic unit (OTU) clustering and species information analysis.

USEARCH was used to cluster OTUs of the above treated sequences at 97% similarity. The representative OTU sequences were compared with the database Silva for species annotation (confidence threshold: 0.6). The relative abundance percentages of each sample were calculated at the phylum, class, order, family, genus and species levels. The rarefaction curve reflects the sequencing depth of the samples. The rank–abundance curve explains species abundance and species evenness.

A Venn diagram can be used to count the common and unique OTU numbers of multiple samples, which can directly show the overlap and uniqueness in the OTU composition of different samples.

Alpha-diversity analysis reflected the richness and diversity of communities in the samples. Mothur (http://www.mothur.org/wiki/Schloss_SOP#Alpha_diversity) was used to calculate the values of the Shannon, Simpson, Chao and ACE indices, and the R (3.4.1) language tool was used for graph plotting.

Jaccard, Bray–Curtis, unweighted UniFrac, and weighted UniFrac were used to calculate the differences between samples and conduct nonmetric multidimensional scaling graphs (NMDS). Metastats (http://metastats.cbcb.umd.edu/) was used for the comparison of the features with different abundances between groups on multiple taxonomic levels. The nonparametric factorial Kruskal–Wallis (KW) sum-rank test was applied to determine the significant difference between the richness of the groups. LEfSe (LDA effect size) uses linear discriminant analysis (LDA) to estimate the impact of each component (species) abundance on the difference effect of the groups.

### In silico prediction of ADMET and drug-likeness properties of BTL-I

To evaluate the pharmacokinetic profile and toxicity of BTL-I, we employed ADMETlab 2.0 (https://admetmesh.scbdd.com/pub/), which is a free online platform that enables researchers to predict the ADMET and drug-likeness properties of a compound [45].

## Results

### BTL-I improved AlCl_3_-induced memory impairment

**Fig. 2:**
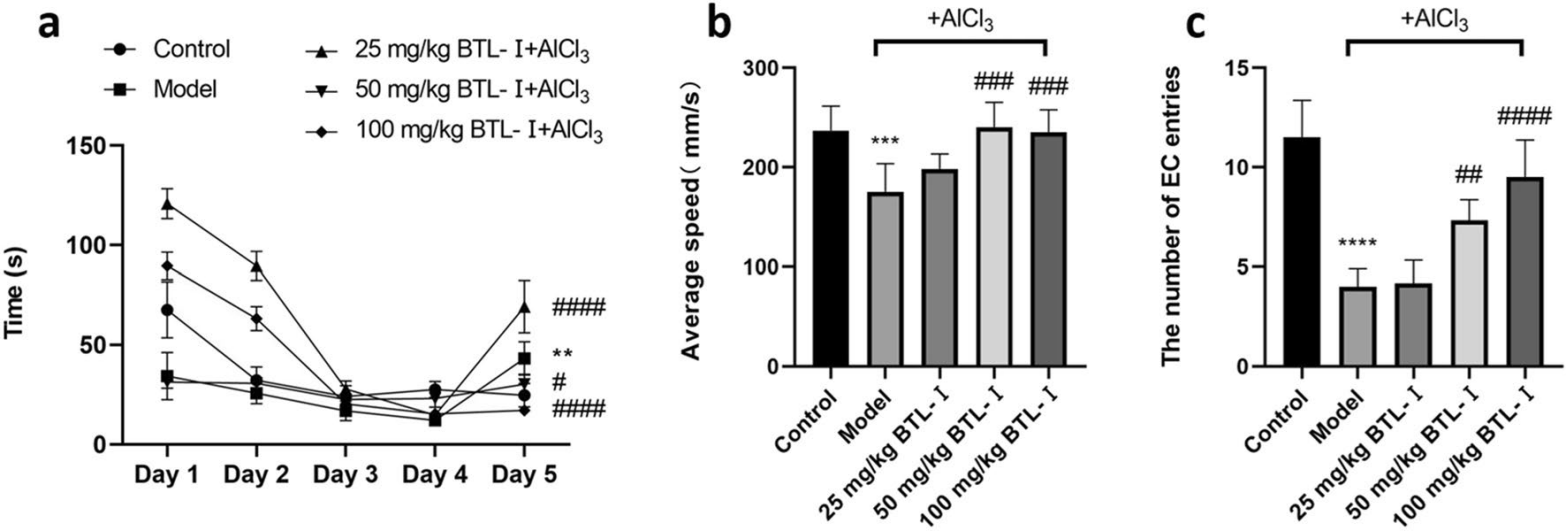
Behavioral performance of zebrafish in the enriched chamber zone of the T-maze test. **a** The latency (s) of first entry into the EC zone of the T-maze test. Day 5: *P < 0.05, **P < 0.01, ***P < 0.005, ****P < 0.001, vs. the control group; ^#^*P* < 0.05, ^##^*P* < 0.01, ^###^*P* < 0.005, ^####^*P* < 0.001, vs. the model group. **b** Average swimming speed on the fifth day (*n* = 6). **c** The number of entries to the EC zone on the fifth day (*n* = 6). *P < 0.05, **P < 0.01, ***P < 0.005, ****P < 0.001, vs. the control group; ^#^*P* < 0.05, ^##^*P* < 0.01, ^###^*P* < 0.005, ^####^*P* < 0.001, vs. the model group

Overall, compared with the model group, the BTL-I-treated groups showed a significant treatment effect on cognitive performance in the T-maze test [*F* (4, 25) = 40.60, *P* < 0.001 for the latency of first entry to the EC zone on day 5 in Fig. 2a, *F* (4, 25) = 9.029, *P* < 0.001 for swimming speed in Fig. 2b, *F* (4, 25) = 31.65, *P* < 0.001 for the numbers of EC entries in Fig. 2c].

**Fig. 3:**
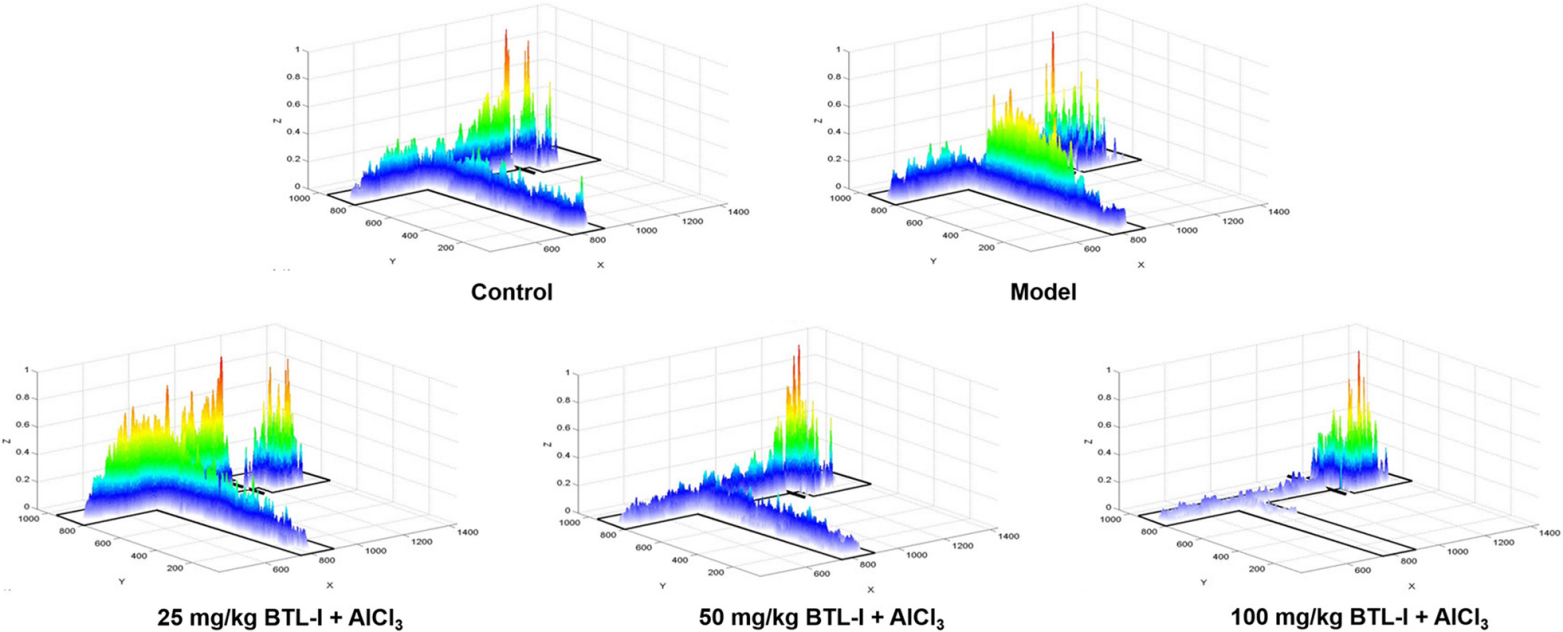
Heatmaps of zebrafish activity in the T-maze on the fifth day. The *X*-axis and *Y*-axis in the figure represent the motion trajectory of zebrafish, while the *Z*-axis represents the residence time of zebrafish. The higher the *Z*-axis is, the longer the residence time of zebrafish at a certain point

Subsequent post hoc testing revealed that zebrafish in the model group had an increased latency of first entry to the EC zone and reduced swimming speed and number of EC entries (Fig. 2b, c). The swimming tracks also clearly showed the reduced preference of the model group fish to the EC zone (Fig. 3) following the AlCl_3_ injections. In contrast, pretreatment with medium and high doses of BTL-I prevented these effects of AlCl_3_.

### Effect of AlCl_3_ on proinflammatory cytokines, AChE and GSH

**Fig. 4:**
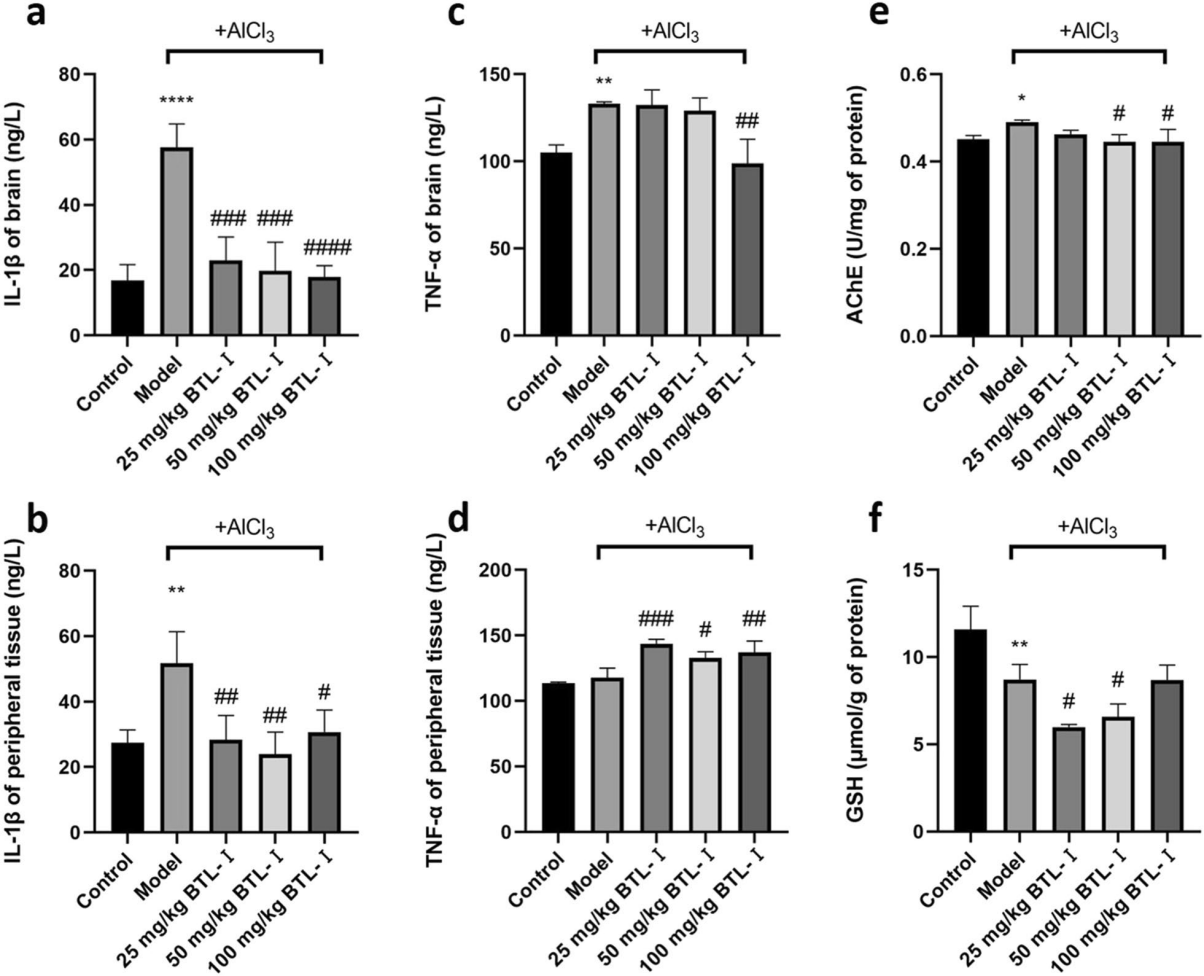
The results of biochemical indices of zebrafish (*n* = 3). **a** GSH content in zebrafish brain tissue. **b** AChE activity in zebrafish brain tissue. **c**–**f** IL-1β and TNF-α content in zebrafish brain and peripheral tissue. *P < 0.05, **P < 0.01, ***P < 0.005, ****P < 0.001, vs. the control group; ^#^*P* < 0.05, ^##^*P* < 0.01, ^###^*P* < 0.005, ^####^*P* < 0.001, vs. the model group

To further elucidate the effect of BTL-I on AlCl_3_-induced cognitive impairment in zebrafish, we assessed changes in the levels of several biochemical indicators (including IL-1β, TNF-α, GSH, AChE). The results showed that AlCl_3_ treatment promoted the release of IL-1β from the brain and peripheral tissue and TNF-α from the brain, and BTL-I supplementation inhibited the release of IL-1β in the brain and periphery, as well as the release of TNF in the brain [*F* (4, 10) = 20.64, *P* < 0.001 for brain IL-1β in Fig. 4a; *F* (4, 10) = 7.240, *P* < 0.01 for peripheral IL-1β in Fig. 4b; *F* (4, 10) = 11.75, *P* < 0.001 for brain TNF-α in Fig. 4c]. BTL-I also increased the peripheral TNF-α level [*F* (4, 10) = 14.94, *P* < 0.001 in Fig. 4d].

Furthermore, paralleling their cognitive deficits in the T-maze, zebrafish treated with AlCl_3_ exhibited higher brain AChE activity, whereas both moderate and high doses of BTL-I dose-dependently inhibited AChE activity [*F* (4, 10) = 4.474, *P* < 0.05 in Fig. 4e]. This is consistent with the fact that excessive AChE activity is closely related to memory deficits [46].

In addition, treatment with AlCl_3_ caused an oxidant–antioxidant imbalance in the brain, and GSH, the key nonenzymatic antioxidant in the body, has important physiological functions, such as scavenging free radicals, detoxifying, promoting iron absorption or maintaining membrane integrity [47–51]. As GSH is a low-molecular-weight scavenger of O_2_^−^, H_2_O_2_ and so on, its content is an important indicator of the antioxidant capacity of the body [52]. Here, AlCl_3_ treatments decreased GSH levels in the fish brain [(*F* (4, 10) = 18.90, *P* < 0.001 in Fig. 4f]. Compared with the model group, the BTL-I treatment groups did not show antioxidant activity since no higher GSH level was observed. However, they dose-dependently increased GSH levels in zebrafish.

### Results of intestinal flora diversity analysis

**Fig. 5:**
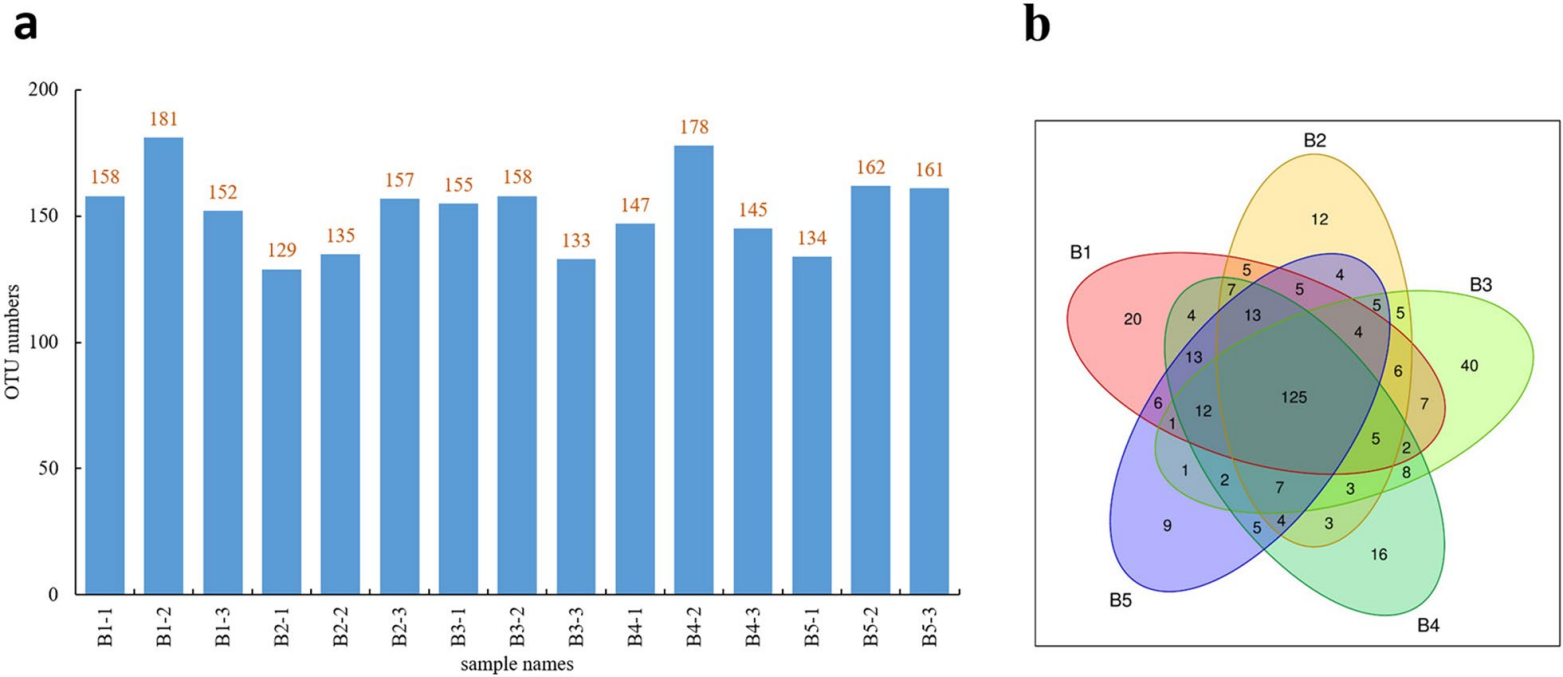
Statistics of the OTUs. **a** Statistics of the OTUs of 15 samples. **b** Differences in the distribution of OTUs between groups using the Venn diagram. Group B1: model, Group B2: control, Group B3: 25 mg/kg BTL-I + AlCl_3_, Group B4: 50 mg/kg BTL-I + AlCl_3_, Group B5: 100 mg/kg BTL-I + AlCl_3_

Moreover, due to the close and inflammation-mediated relationship between gut microbiota (GMB) and NDs, the change of intestinal flora of zebrafish is another important indicator for our determination. OTUs are hypothetical computational taxa (e.g., strain, species, genus, group) that have been artificially established to facilitate the analysis of phylogenetic or population genetics. Because of the conservatism of 16S rDNA, the sequence obtained by sequencing can represent a species. To understand the composition of a species in a population sample, it is necessary to cluster the sequences. By clustering, the sequence is divided into many groups according to similarity, and one group is an OTU [53]. In this study, 15 samples were investigated, and the number of OTUs received by each sample is shown in Fig. 5a.

A Venn diagram was used to count the common and unique OTU numbers of multiple samples, which can intuitively show the similarity and overlap of the OTU number composition of specific samples. Figure 5b shows the differences in OTUs between the five groups. Different colors represent different groups, and the intersecting part is the OTU shared by adjacent groups. The OTUs in each group were as follows: Group B1 (model) 235; Group B2 (control) 213; Group B3 (25 mg/kg BTL-I + AlCl_3_) 233; Group B4 (50 mg/kg BTL-I + AlCl_3_) 229; and Group B5 (100 mg/kg BTL-I + AlCl_3_) 216. The OTUs common between the model group and the other groups were as follows: 163 (Groups B1 and B2); 160 (Groups B1 and B3); 181 (Groups B1 and B4); and 179 (Groups B1 and B5) (Fig. 5b).

**Fig. 6:**
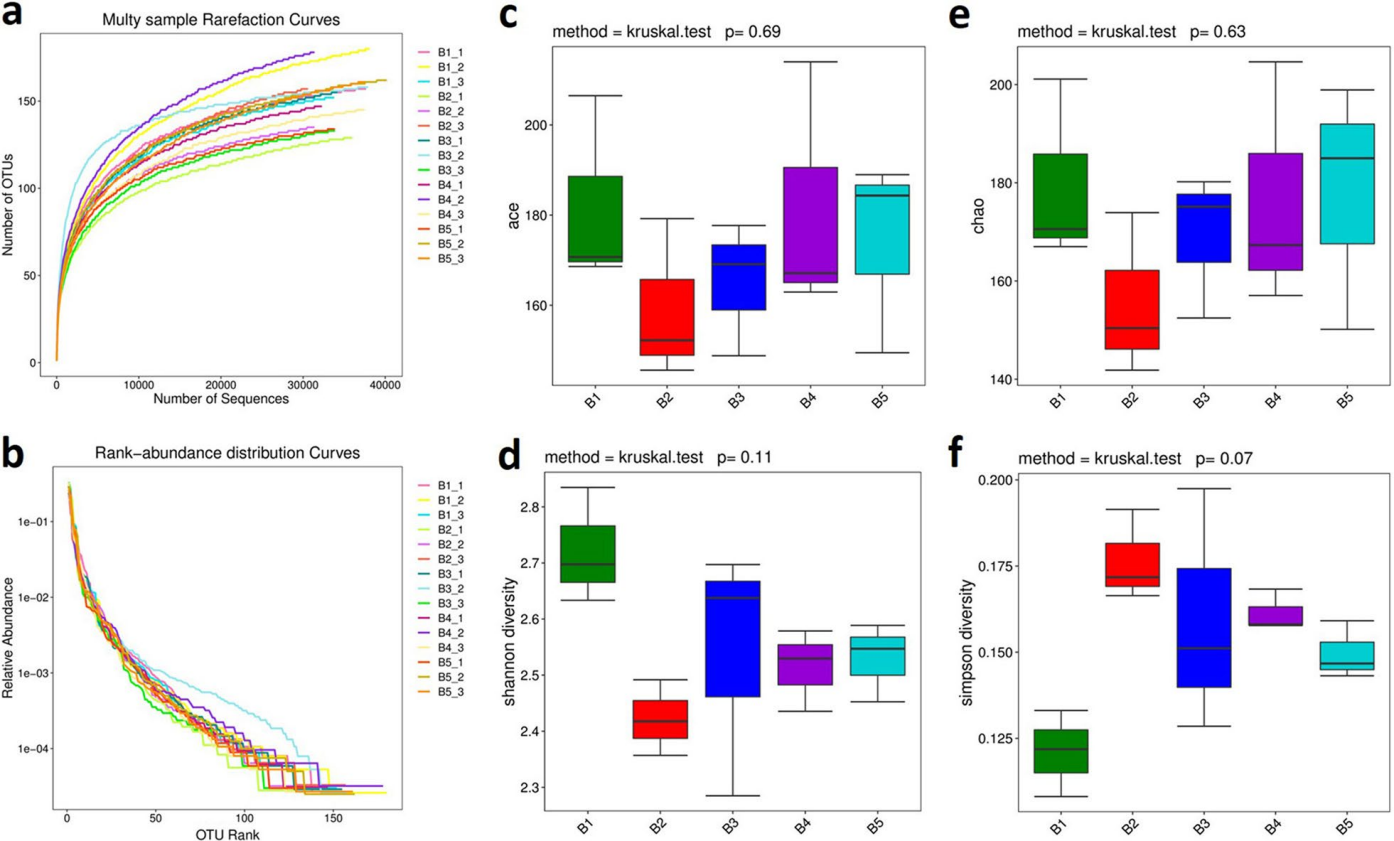
Alpha-diversity. **a** The rarefaction curves. **b** The rank–abundance curves. **c**–**f** Alpha indices

To assess the rationality of intestinal flora sequencing of samples, we constructed rarefaction curves and ranked abundance curves of intestinal flora according to the OTU numbers at different sequencing depths. The curve tends to be flat from 10,000 reads, indicating that the sequencing data volume is adequate, and a greater data volume will only produce a small number of new OTUs (Fig. 6a). A rank–abundance curve can be used to explain the abundance and evenness of species. In the horizontal direction, the abundance of species is reflected by the width of the curve (i.e., the higher the abundance of species is, the wider the range of the curve is). The shape (smoothness) of the curve reflects the evenness of species in the sample (i.e., the flatter the curve, the more uniform the distribution of species). The results showed that the rank–abundance curve was smooth except for individual samples, indicating that the species distribution of each sample was even (Fig. 6b).

Alpha-diversity can reflect the abundance and diversity of microbial communities, including the Chao index, Ace index, Shannon index, and Simpson index. The Chao and Ace indices reflect the species richness, i.e., the number of species in the sample, without considering the abundance of each species. Shannon and Simpson indices reflect both species richness and species evenness in the community. The comparison between all five groups showed that there was no significant total difference in bacterial diversity. However, the Shannon and Simpson indices displayed relatively larger differences between the model and control groups than between the control and administration groups (Fig. 6c–f).

Beta diversity analysis was performed to compare the differences in species diversity of the paired samples. The contents of each species in the samples were analyzed, and the beta diversity values among different samples were then calculated.

**Fig. 7:**
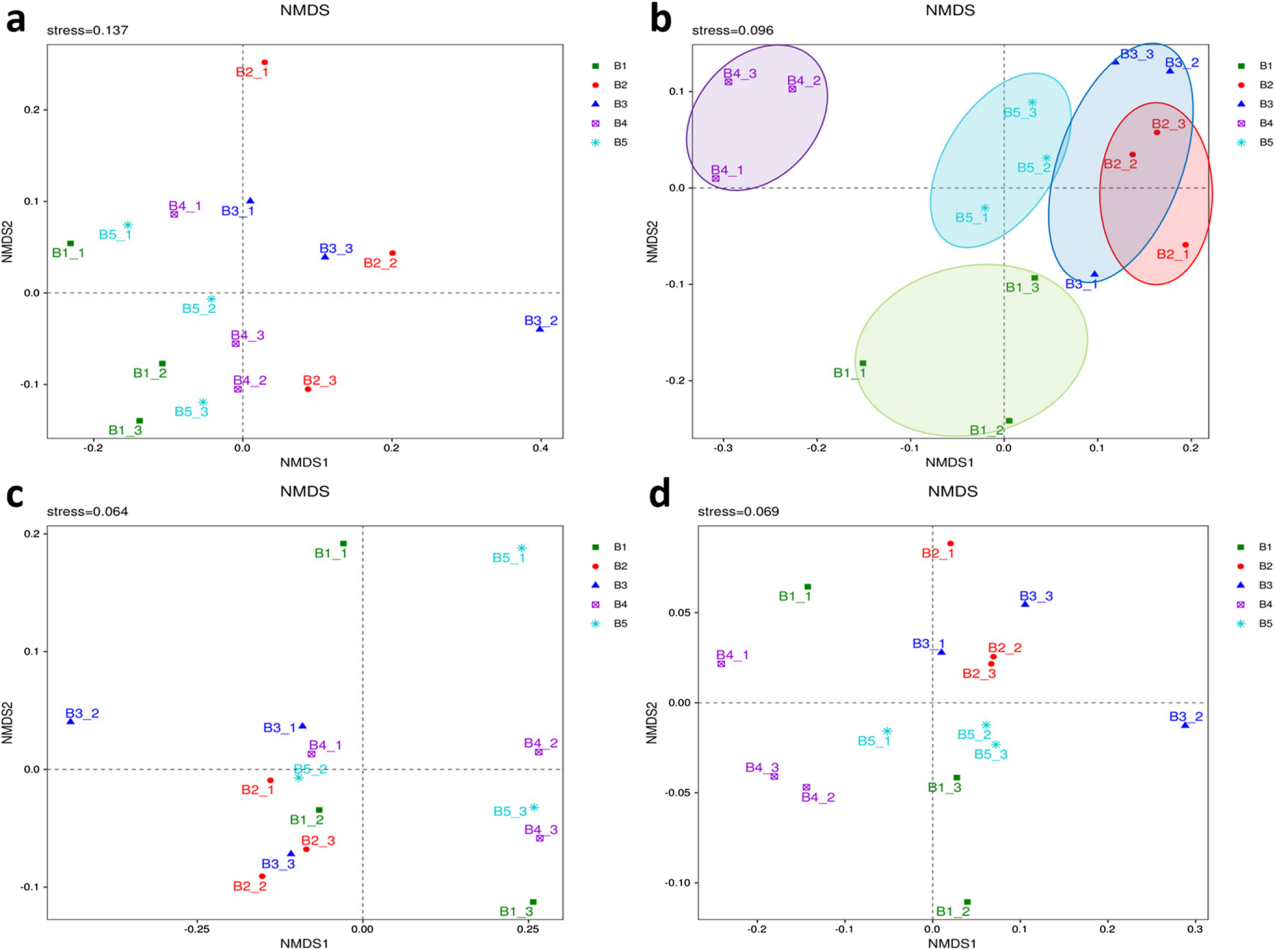
Beta diversity. **a** Jaccard algorithm (stress value = 0.137). Only the presence or absence of OTUs in the sample was considered, not the abundance. **b** Bray–Curtis algorithm (stress value = 0.096). Both the presence and absence of OTUs in the sample and the abundance were considered. **c** Unweighted-UniFrac algorithm (stress value = 0.064). It only considers whether the sequence was present in the community, not the abundance of the sequence. **d** Weighted-UniFrac algorithm (stress value = 0.069). It accounts for the abundance of sequences on the basis of unweighted UniFrac and was able to differentiate species abundance

The NMDS method is a data analysis method that simplifies the research objects in multidimensional space to low-dimensional space for positioning, analysis and classification while retaining the original relationship between objects. The degree of difference between samples was reflected by the distance between points. Four algorithms, Jaccard, Bray–Curtis, unweighted UniFrac, and weighted UniFrac, were used for NMDS calculation. The NMDS based on the Jaccard algorithm only considers whether a specific OTU existed in the sample, not its abundance. The NMDS based on the Bray–Curtis algorithm considers both OTU varieties and abundances in samples. UniFrac analysis uses evolutionary information of sample sequences to compare whether the samples have significant microbial community differences in a particular evolutionary lineage. The unweighted UniFrac method only considers whether the specific sequence appears in the community, not its abundance. The weighted UniFrac method takes both existence and abundance into account. The results of the Jaccard, unweighted UniFrac, and weighted UniFrac methods showed that there was no significant difference in OTU varieties or evolutionary lineage between the experimental groups, the control group and the model group (Fig. 7a, c, d). However, the results of the Bray–Curtis method showed that there were significant differences in OTU abundance between the model and control groups, whereas there was no significant difference in OTU abundance between the experimental groups (except for the 50 mg/kg group) and the control group (Fig. 7b).

**Fig. 8:**
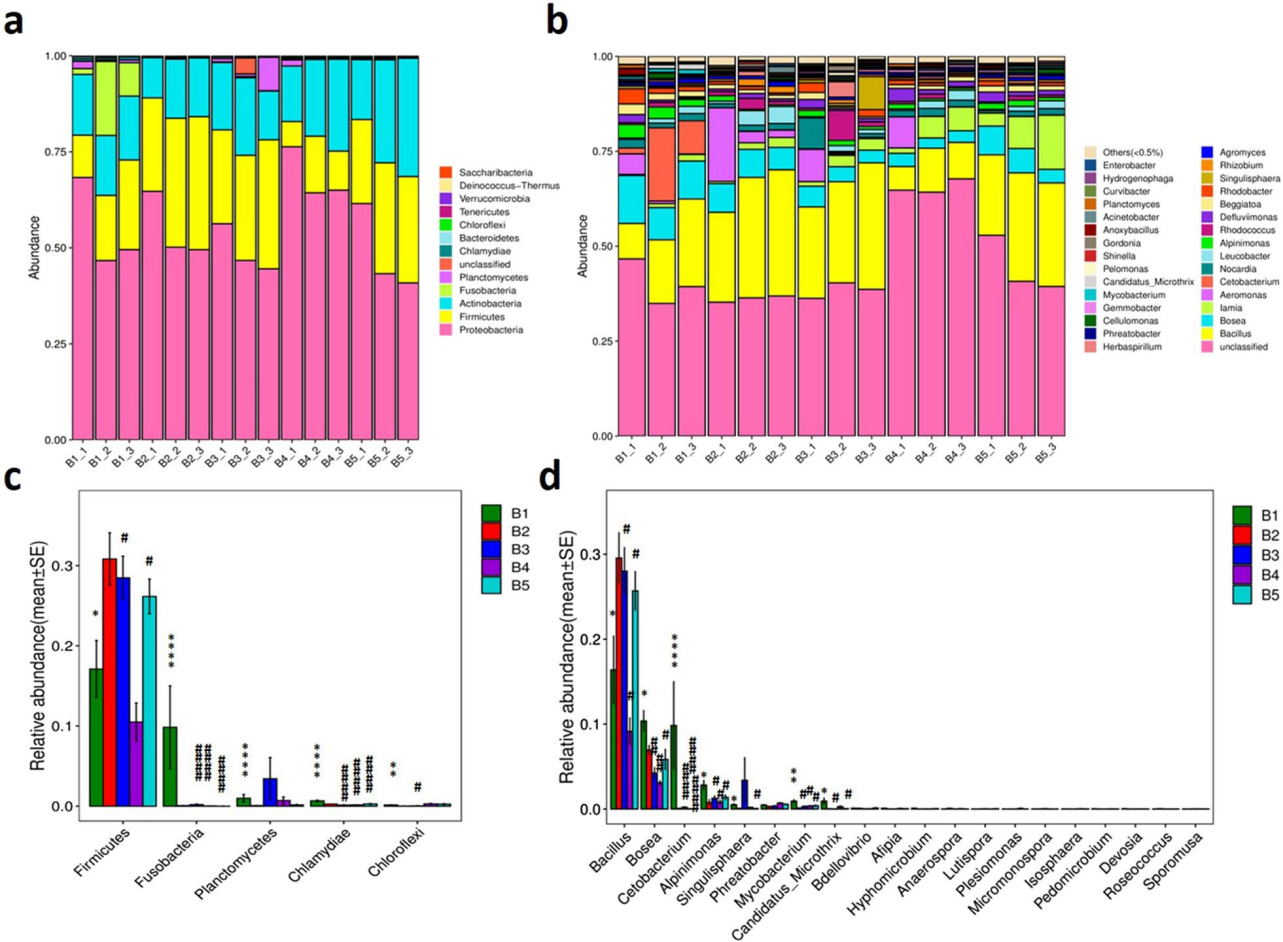
Relative abundance (**a** and **b**) and analysis of differential microorganisms (**c** and **d**). **a** and **c** Results at the phylum level. **b** and **d** Results at the genus level. *P < 0.05, **P < 0.01, ***P < 0.005, ****P < 0.001, vs. the control group; ^#^*P* < 0.05, ^##^*P* < 0.01, ^###^*P* < 0.005, ^####^*P* < 0.001, vs. the model group

Microbial diversity analysis showed that the intestinal flora of zebrafish included the following 12 major phyla: Proteobacteria, Firmicutes, Actinobacteria, Fusobacteria, Planctomycetes, Chlamydiae, Bacteroidetes, Chloroflexi, Tenericutes, Verrucomicrobia, Deinococcus-Thermus and Saccharibacteria. Among them, Proteobacteria, Firmicutes and Actinobacteria were the dominant bacteria at the phylum level (Fig. 8a). The abundances of Firmicutes in the gut of the model group were reduced, whereas those of Fusobacteria, Planctomycetes, Chlamydiae and Chloroflexi significantly increased compared with those observed in the control group. In the two experimental groups (administration of BTL-I 25 and 100 mg/kg), the abundance of Firmicutes was significantly increased, whereas those of Fusobacteria and Chlamydiae significantly decreased compared with that in the model group and basically returned to the same level as that in the control group. However, in the BTL-I treatment groups, there was almost no significant reversal effect on the increase in Planctomycetes and Chloroflexi abundance (Fig. 8c).

At the genus level, a total of 30 major known taxa of intestinal flora were identified (Fig. 8b). The first 8 genera with intergroup abundance differences were *Bacillus*, *Bosea*, *Cetobacterium*, *Alpinimonas*, *Singulisphaera*, *Phreatobacter*, *Mycobacterium*, and *Candidatus*-*Microthrix* (Fig. 8d). Compared with the control group, the abundance of *Bacillus* in the model group significantly decreased, while those of the other seven genera mostly increased significantly. In the two BTL-I treatment groups (25 and 100 mg/kg), the abundance of *Bacillus* was elevated to nearly normal levels, while those of *Bosea* and the other genera mostly decreased significantly.

**Fig. 9:**
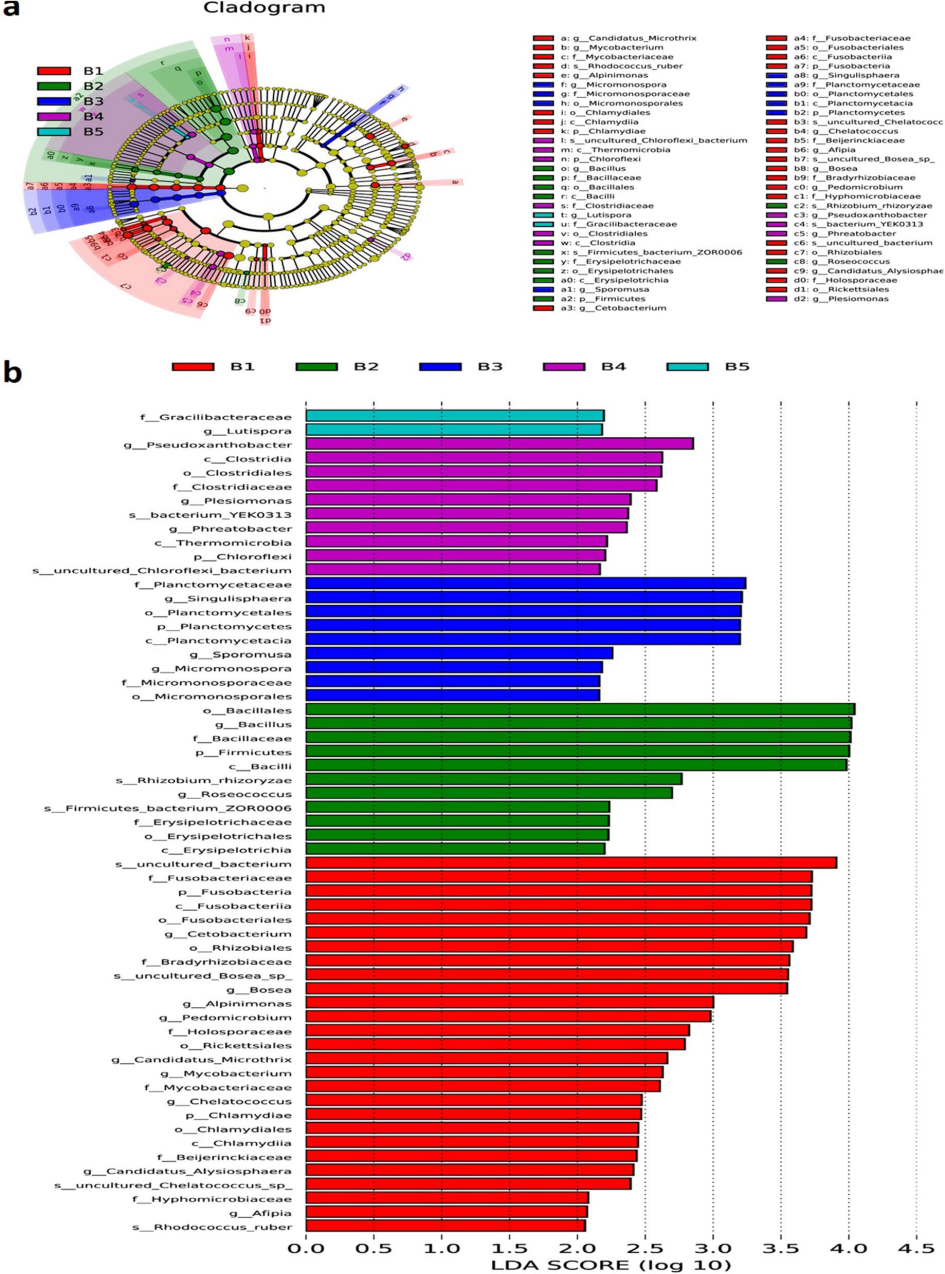
LDA effect size analysis. **a** Branching diagram of the evolution of different species between the control, model and experimental groups. **b** Bar graph of LDA values for different species

LEfSe was able to compare the taxonomic composition of multiple groups at different taxonomic levels, identify the taxa with significant intergroup differences in abundance (i.e., biomarkers), and exhibit their lineage relationship. The results in Fig. 9 show the biomarkers with significant effects (LDA scores > 2) in each group, including 27 taxa in the model group (e.g., *Cetobacterium* in Fusobacteria, *Bosea* in Rhizobiales, Chlamydiales, *Candidatus-Microthrix*, *Mycobacterium*), 11 taxa in the control group (e.g., *Bacillus* in Firmicutes, *Rhizobium rhizoryzae*), 9 taxa in the low-dose group (25 mg/kg BTL-I) (e.g., *Singulisphaera* in Planctomycetes, *Micromonospora*), 10 taxa in the medium-dose group (50 mg/kg BTL-I) (e.g., Clostridiaceae, Chloroflexi, *Pseudoxanthobacter*), and 2 taxa in the high-dose group (100 mg/kg BTL-I) (Gracilibacteraceae and *Lutispora*).

### Prediction of ADMET and drug-likeness properties

Finally, the early evaluation of lead compound for its potential to become a drug is critical step for drug development. To obtain more information on the pharmacokinetic profile of BTL-I and whether it has the potential to become a drug, we used ADMETlab 2.0 [45] to predict its ADMET and drug-likeness properties. The corresponding predicted results are presented in Table 1, and the physical properties of BTL-I are shown in Additional file 1: Table S1. The results demonstrated that BTL-I possesses acceptable ADMET and drug-likeness properties in general. For example, the results showed that BTL-I is active in both human intestinal absorption (HIA) and blood–brain barrier (BBB) penetration. It has acceptable safety profiles, generally performing well on most metrics (e.g., hERG blockers, Ames toxicity and carcinogenicity), and it is in harmony with the Lipinski rule [54] and others (such as the Pfizer rule [55] and golden triangle [56]), which indicates the drug-likeness properties of a compound. Unfortunately, BTL-I displayed some disadvantages, such as a high risk of inhibiting CYP2C19, CYP2C9 and CYP3A4 and inducing liver injury.

**Table 1:** ADMET and drug-likeness properties of BTL-I through the online prediction tool ADMETlab 2.0 (the table is located below line 394)

Property	Value	Decision
Absorption		
Caco-2 permeability	− 4.9 log cm/s	Excellent
Madin–Darby canine kidney cells (MDCK) permeability	2e-05 cm/s	Excellent
P-glycoprotein (Pgp)-inhibitor	0.023	Excellent
P-glycoprotein (Pgp)-substrate	0.007	Excellent
Human intestinal absorption (HIA)	0.008	Excellent
20% bioavailability (F20%)	0.059	Excellent
Distribution		
Plasma protein binding (PPB)	0.987	Bad
Volume distribution (VD)	0.501 L/kg	Excellent
Blood–brain barrier (BBB) penetration	0.027	Excellent
The fraction unbound in plasma (Fu)	0.012	Bad
Metabolism		
CYP1A2-inhibitor	0.381	–
CYP1A2-substrate	0.536	–
CYP2C19-inhibitor	0.955	–
CYP2C19-substrate	0.189	–
CYP2C9-inhibitor	0.934	–
CYP2C9-substrate	0.946	–
CYP2D6-inhibitor	0.873	–
CYP2D6-substrate	0.788	–
CYP3A4-inhibitor	0.916	–
CYP3A4-substrate	0.333	–
Excretion		
Clearance	17.179 mL/min/kg	Excellent
The half-life (T_1/2_)	0.371	–
Toxicity		
hERG blockers	0.023	Excellent
Human hepatotoxicity (H-HT)	0.402	Medium
Drug-induced liver injury (DILI)	0.764	Bad
Ames toxicity	0.106	Excellent
Rat oral acute toxicity	0.559	Medium
Maximum recommended daily dose (FDAMDD)	0.283	Excellent
Skin sensitization	0.105	Excellent
Carcinogenicity	0.162	Excellent
Eye corrosion	0.003	Excellent
Eye irritation	0.146	Excellent
Respiratory toxicity	0.039	Excellent
Drug-likeness		
MCE-18 [56]	68.839	Excellent
Lipinski rule [53]	Accepted (0 violation)	Excellent
Pfizer rule [54]	Accepted (0 violation)	Excellent
Golden triangle [55]	Accepted (0 violation)	Excellent
GSK rule [57]	Rejected (1 violation)	Bad

## Discussion

Aluminum has been examined for its broad neurotoxic effects and close relationship with AD, which promote tau hyperphosphorylation, aggregation, and neurofibrillary tangle formation in AD brains (by activating tau kinases CDK5 and GSK3β), accumulate in microglia and induce proinflammatory cytokines, bind to Aβ and induce its aggregation, stimulate iron-induced membrane lipid peroxidation and oxidative damage, decrease the activity of antioxidant enzymes, and interact with AChE on γ-peripheral sites to enhance enzymatic activity, resulting in reduced neurotransmission [12, 59, 60]. Furthermore, activated AChE can deteriorate Aβ aggregation, decrease BDNF expression [59], and further promote oxidative stress and neuroinflammation through a ‘cholinergic anti-inflammatory pathway’ (CAIP) via α7 nicotinic acetylcholine receptors [60, 61]. In addition, in many reports on aluminum-induced AD or toxicity models, alterations in the host gut microbiota have been observed [62, 63].

Therefore, the present study established a zebrafish model of subchronic inflammation induced by acute i.p. AlCl_3_ administration, which resulted in subchronic peripheral and central inflammatory responses and enhanced oxidative stress and AChE activity in the brain. At the behavioral level, administration of AlCl_3_ strongly impaired the spatial and contextual memory of zebrafish in the T-maze test. At the gut microbiota level, high-throughput sequencing results showed that the intestinal flora of zebrafish was dramatically disturbed by acute AlCl_3_ administration. Collectively, these findings are generally consistent with previous evidence that AlCl_3_ induces memory deficits in both humans and animals, including zebrafish, and changes the intestinal flora [62–64]. In contrast, BTL-I co-administration reversed these induced memory deficits and microbiota imbalances, indicating the potential neuroprotective role of this drug.

In the present study, acute central and peripheral inflammation was characterized by the release of the proinflammatory cytokines IL-1β and TNF-α following AlCl_3_ administration. Supplementation with BTL-I potently inhibited acute brain and peripheral inflammation in AlCl_3_-treated zebrafish. Mounting evidence implicates BTL-I in multitargeted neuroprotective activity against oxidative stress, neuroinflammation and neuronal apoptosis, as well as in nerve growth without inducing cytotoxicity [23–27]. Here, we found that BTL-I also prevents cognitive deficits (induced in zebrafish by AlCl_3_) and exerts neuroprotective effects in this zebrafish model.

In this study, we noted that the peripheral IL-1β level was suppressed while TNF-α level were elevated after BTL-I administration. The different behaviors of the two inflammation markers are consistent with Tsarouchas’ report about the effects of spinal cord injury on zebrafish spinal axonal regeneration that increased TNF-α (mainly produced by macrophage) and decreased IL-1β (mainly produced by neutrophil) both promote neurite regeneration in the periphery [65]. So, we presume that the upregulated peripheral TNF-α and downregulated IL-1β in BTL-I-treated zebrafish are actually consistent with the improvement of cognition. However, further investigation is necessary to reveal the exact mechanism.

Meanwhile, the inhibition of brain AChE elevates ACh levels and, hence, positively affects cognitive function in rats [60, 66]. Subchronic exposure of zebrafish to AlCl_3_ or i.p. injection of AlCl_3_ in mice enhances brain AChE activity [39]. Accordingly, our results show that i.p. injection of AlCl_3_ also elevated AChE activity in the zebrafish brain, whereas BTL-I evoked neuroprotection and lowered AChE activity (Fig. 4e). Because thin-layer chromatography bioautography shows that BTL-I does not inhibit AChE catalytic activity (data not shown), this compound seems to indirectly decrease zebrafish AChE activity here, likely involving other molecular pathways.

Furthermore, oxidative stress involves the excessive production of ROS and reactive nitrogen species (RNS) [67] and may result in tissue damage. GSH is the most important nonenzymatic antioxidant, the neuroprotective role of which in the brain is critical against oxidative damage caused by catecholamine oxidation or lipid peroxidation [68]. In the present study, GSH levels markedly decreased 24 h after AlCl_3_ administration. With BTL-I pretreatment at doses of 25 and 50 mg/kg, the GSH levels of the zebrafish were even lower than those of the model group. However, when the dose increased to 100 mg/kg, the GSH content increased to the same level as that in the model group, although it was still lower than that in the control group. BTL-I seemed to display doubtful antioxidant effects via GSH. The lack of significant antioxidant effects of BTL-I relative to the model group may be due to the limited sample size and the resulting low statistical power. However, it may also suggest that the antioxidant mechanism of action of BTL-I may exist elsewhere. Further studies with larger sample sizes and better designs are warranted to test and explain this complicated phenomenon to obtain a solid conclusion.

In addition, the gut microbiota (GMB) plays a crucial role in the stability and balance of the intestinal microecological environment, and the composition of the human intestinal microbial community remains basically stable after the age of 3 years [69]. In recent years, a growing number of studies have shown that dysregulation of specific GMB is closely related to NDs [15, 16, 70]. Some reports have indicated that in the gut of healthy humans or animals, there are higher populations of Gram-positive (G^+^) bacteria, including Firmicutes and Actinobacteria, and lower populations of Gram-negative (G^−^) bacteria, such as Bacteroidetes, at the phylum level [15, 16]. At the family or genus level, some G^+^ taxa, such as *Bacillus*, *Eubacterium*, Clostridiaceae in Firmicutes and *Bifidobacterium* in Actinobacteria, show a higher abundance in healthy individuals and benefit their hosts through different mechanisms, including reducing leakage of gut by the protection of biofilms, inhibiting inflammation and antioxidation and reducing Aβ deposition and transfer from the gut to the brain [15–17]. In contrast, some G^−^ taxa*,* such as *Bacteroides*, *Blautia*, *Escherichia coli*, *Shigella*, *Chlamydia*, and *Fusobacterium*, are closely and positively correlated with AD, mainly involving the activation of systematic inflammation by their enriched LPS in the cell wall and the invasion of proinflammatory cytokines, LPS, and even bacteria into the blood circulation system and brain, inducing Aβ deposition and tau phosphorylation [15–17, 70].

In the present study, the control group zebrafish hosted a greater amount of G^+^ bacteria (Firmicutes at the phylum level and predominantly *Bacillus* at the genus level) than the AlCl_3_-injured model group with memory impairment, while the model group zebrafish had much fewer G^+^ bacteria than the control group zebrafish but significantly more G^−^ bacteria, including *Cetobacterium* (in the family Fusobacteriaceae) and Chlamydiales (at the order level). This result is highly consistent with previous studies, especially reports on the benefits of *Bacillus subtilis* in delaying neurodegeneration and behavior impairment in the AD model *Caenorhabditis elegans* and reports on the negative effect of G^−^ bacteria, including *Fusobacterium* and *Chlamydia* [17, 70, 71].

Generally, pretreatment with BTL-I maintained the dominance of G^+^ bacteria vs. G^−^ bacteria in the gut of zebrafish when faced with aluminum exposure. However, the dose levels exerted different influences. In the low- and high-dose groups, the abundances of *Bacillus* remained at high levels close to those of the control group, and the abundances of G^−^ bacteria were much lower. Additionally, in the low-dose group, another G^+^ bacterium, *Micromonospora,* in the phylum Actinobacteria was recognized as a biomarker, suggesting its possible positive role based on a report [16] on Actinobacteria. Likewise, in the high-dose group, the G^+^ bacteria *Lutispora* in the family Gracilibacteriaceae and the order Clostridiales were also biomarkers. Considering the report on the strongly negative correlation of Clostridiaceae [16] with AD biomarkers in cerebrospinal fluid, we speculate that *Lutispora* may also have some benefit in neuroprotection.

It is intriguing that the middle-dose group did not possess a high abundance of *Bacillus*. This discrepancy may be attributable to a nonlinear relationship of BTL-I and *Bacillus,* and more concentration gradients of BTL-I will be set in future studies to explore this relationship. However, the G^+^ bacterium Clostridiaceae was found to be the key biomarker of this group; G^−^ bacterium Chloroflexi was another key biomarker, but bacteria in this phylum have no LPS in their cell walls [72]. These findings may help to explain the behavioral improvement of this group.

Our study suggests that administration of the marine fungal metabolite BTL-I prior to AlCl_3_ injection may aid in maintaining the predominance of beneficial G^+^ bacteria in the gut of zebrafish to resist acute injury caused by aluminum, related inflammation and AD pathology. We deduce that the stabilization of the GMB may contribute to the clearance of inflammation and lead to the improvement of zebrafish’s cognition. However, the detailed mechanisms of intestinal flora regulation and the treatment effect on an AlCl_3_-induced chronic AD model need to be further investigated for BTL-I in the future.

Further, the early evaluation of ADMET and drug-likeness properties of drug candidates are highly significant, as many drugs have been withdrawn in clinical trials and even in the marketing process due to unacceptable pharmacokinetic properties [73–75]. Accordingly, the prediction of ADMET and drug-likeness properties of drug candidates has received extensive attention. Numerous tools have been developed, such as ADMETlab [76] admetSAR [77] and SwissADME [78]. In this study, in silico prediction with ADMETlab suggested that BTL-I caters to the majority of the ADMET properties and drug-likeness profiles, such as the typical Lipinski rule with 0 violation, and possesses good properties in crossing the BBB. Such features render it a promising drug candidate for NDs since overcoming BBB penetration is essential for NDs drugs [79, 80]. It should be noted that BTL-I was predicted to be associated with a high risk of liver injury, which requires further confirmation and assessment and may provide clues for structural optimization. In addition, it is predicted that CYP2C19, CYP2C9 and CYP3A4 may be inhibited by BTL-I and therefore co-administration with CYP2C19, CYP2C9 and CYP3A4-substrate drugs may be avoided.

## Conclusions

This study showed that BTL-I dose-dependently ameliorated AlCl_3_-induced cognitive deficits in zebrafish, reversed the elevation of AlCl_3_-induced central and peripheral proinflammatory cytokine levels and the increase in brain AChE activity, and contributed to maintaining the predominance of beneficial Gram-positive bacteria in the GMB of zebrafish, which was challenged by AlCl_3_. The in silico analysis indicated that BTL-I exhibits acceptable drug-likeness and ADMET profiles. In summary, BTL-I has the potential as an intervention agent for preventing CNS deficits caused by inflammation, neurotoxicity, and GMB imbalance.

## Supplementary Information

**Additional file 1.** Physicochemical properties of BTL-I through online prediction tool of ADMETlab 2.0.

## Data Availability

All data generated or analyzed during this study are included in this published article and its additional file. The datasets used and/or analyzed during the current study are available from the primary author on reasonable request.

## Abbreviations

AChE: Acetylcholinesterase
AD: Alzheimer’s disease
ADMET: Absorption, distribution, metabolism, excretion, and toxicity
AlCl_3_: Aluminum trichloride
Aβ: β-Amyloid peptide
BTL-I: Butyrolactone I
BTLs: Butyrolactones
CAIP: Cholinergic anti-inflammatory pathway
CDK 5: Cyclin-dependent kinase 5
CNS: Central nervous system
COX-2: Cyclooxygenase-2
GMB: Gut microbiota
GSH: Glutathione
IL-1β: Interleukin-1β
KW: Kruskal–Wallis
LDA: Linear discriminant analysis
NDs: Neurodegenerative disorders
NMDS: Nonmetric multidimensional scaling graphs
PD: Parkinson’s disease
RNS: Reactive nitrogen species
ROS: Reactive oxygen species
TNF-α: Tumor necrosis factor-α
