# Supplementary material for: Marine fungal metabolite butyrolactone I prevents cognitive deficits by relieving inflammation and intestinal microbiota imbalance on aluminum trichloride-injured zebrafish
Source: J Neuroinflammation. 2022 Feb 7;19:39. doi: 10.1186/s12974-022-02403-3 (PMC8822793; doi:10.1186/s12974-022-02403-3)
Supplement: Supplementary file 1 — Additional file 1. Physicochemical properties of BTL-I through online prediction tool of ADMETlab 2.0. [file 12974_2022_2403_MOESM1_ESM.docx]

**Additional file 1:** **Table S1** **Physicochemical properties of BTL-I through online prediction tool of ADMETlab 2.0.**

| Property | Value | Comment |
| --- | --- | --- |
| Molecular weight | 424.15 g/mol | Optimal: 100~600 g/mol |
| Volume | 433.158 cm^3^/mol | - |
| Density | 0.979 g/cm³ | - |
| Number of hydrogen bond acceptors | 7 | Optimal: 0~12 |
| Number of hydrogen bond donors | 2 | Optimal: 0~7 |
| Number of rotatable bonds | 7 | Optimal: 0~11 |
| Number of rings | 3 | Optimal: 0~6 |
| Number of atoms in the biggest ring | 6 | Optimal: 0~18 |
| Number of heteroatoms | 7 | Optimal: 1~15 |
| Formal charge | 0 | Optimal: -4~4 |
| Number of rigid bonds | 21 | Optimal: 0~30 |
| Flexibility | 0.333 | - |
| Stereo centers | 2 | Optimal: ≤ 2 |
| Topological polar surface area (TPSA) | 110.13 Å² | Optimal: 0~140 Å² |
| Log of the aqueous solubility (LogS) | -4.275 log mol/L | Optimal: -4~0.5 log mol/L |
| Log of the octanol/water partition coefficient (LogP) | 3.754 log mol/L | Optimal: 0~3 |
| LogP at physiological pH 7.4 (LogD) | 3.14 log mol/L | Optimal: 1~3 |
